# Preventive Psychological Interventions for the Management of Perinatal Anxiety: A Systematic Review

**DOI:** 10.3390/brainsci15080861

**Published:** 2025-08-13

**Authors:** Alba Val, Cristina M. Posse, M. Carmen Míguez

**Affiliations:** Department of Clinical Psychology and Psychobiology, Faculty of Psychology, Institute of Psychology (IPsiUS), University of Santiago de Compostela, Campus Vida, 15782 Santiago de Compostela, Spain; alba.val@rai.usc.es (A.V.); cristinamaria.posse.cepeda@usc.es (C.M.P.)

**Keywords:** anxiety, perinatal, pregnancy, preventive, psychological interventions

## Abstract

Introduction: Anxiety is a common problem during pregnancy and postpartum that can have important consequences for mothers and their babies. Having preventive psychological interventions to apply during the perinatal stage could help to reduce its adverse effects. The aim of this study was to find out which psychological interventions have been applied for the prevention of perinatal anxiety, what therapeutic approach and application format have been most commonly used, and which interventions have proven to be most effective. Methods: A literature review was conducted in the PsycInfo, Medline, and SCOPUS databases to identify articles published between March 2015 and March 2025. Results: Twenty studies were selected that met the inclusion criteria. Twelve of the interventions analyzed were indicated prevention programs and eight were universal prevention programs, with most taking place in pregnancy (*n* = 18). Mindfulness and cognitive behavioral therapy were the most commonly employed approaches. Regarding the application format, interventions conducted face-to-face and online were equally frequent, as well as those carried out individually or in groups. The duration ranged from 4 to 14 sessions. Cognitive behavioral therapy interventions, applied face-to-face and in groups, proved to be the most effective. Conclusions: Preventive psychological interventions are effective in reducing anxiety during pregnancy. Further research is needed to draw conclusive results on their long-term effects and efficacy in the postpartum period.

## 1. Introduction

The perinatal period represents a time when the risk of developing a mental health problem increases [1,2] or exacerbating a pre-existing condition [3]. An estimated 20% of women may develop a mental disorder during the perinatal stage, primarily anxiety and depression [4,5]. In recent years there has been a growing interest in exploring the prevalence and consequences of anxiety in the perinatal period (e.g., [6,7,8]), leading to the knowledge that anxiety is a common mental health problem during the perinatal stage [6]. The rates of anxiety disorders and symptoms during pregnancy are between 15.0% and 23.0%, and between 9.0% and 15.0% during postpartum [6,9,10].

Untreated perinatal anxiety leads to adverse consequences for both mothers and their children [11]. Regarding mothers, it has been associated with an increased likelihood of developing postpartum depression [12], an increased risk of preeclampsia, obstetric complications, and bonding problems [13,14]. As for newborns, it has been observed that they are more likely to have lower birth weight and poor cognitive development, among others [15,16,17]. These consequences can result in increased use of educational and healthcare resources [18], which translates into higher economic costs for society [19]. The above justifies the importance of early detection and intervention on anxiety to promote the well-being of mothers and children.

While there is increasing interest in maternal mental health, most research has focused on assessing and treating perinatal depression [8,20,21]. However, the literature on effective treatment and clinical management of perinatal anxiety through psychological interventions is limited [22]. An example of this is the absence of a review on the subject in the Cochrane Library, as opposed to the existence of a specific review on the prevention of postpartum depression [23]. It is important to keep in mind that in this period women prefer psychological interventions [24] and, in addition, a non-pharmacological approach should be adopted because of the potential risks of medication use during pregnancy, such as miscarriages, birth malformations or breastfeeding difficulties, among others [25,26,27].

When researching existing psychological interventions to address perinatal anxiety, it is important to clarify the difference between interventions aimed at prevention and those aimed at treatment. The latter refer to interventions aimed at reducing or eliminating a disease once it has begun, although ideally it would be possible to prevent its onset. Several approaches have shown promise for the treatment of perinatal anxiety, such as cognitive behavioral therapy [28], interpersonal therapy [8] or mindfulness-based therapies [29]. On the other hand, preventive interventions refer to actions aimed at avoiding the onset and consequences of a disease. In the context of perinatal anxiety, prevention could avoid possible adverse health consequences for women and their babies (e.g., increased likelihood of postpartum depression, obstetric complications, bonding problems, lower birth weight, poorer cognitive development). Prevention also has the potential to reduce health care costs [30] and barriers that prevent people from receiving treatment, such as the lack of mental health professionals [31] to meet the growing mental health care needs of this population. Thus, prioritizing prevention could reduce the burden on health systems.

Preventive interventions are classified according to their scope. Thus, universal prevention interventions target the entire population, whereas indicated prevention interventions focus on a high-risk population [32]. Both universal and indicated approaches have been effective in preventing mental health problems in perinatal populations. For example, interventions to prevent perinatal depression have been shown to be effective [33] and are recommended by the United States Preventive Services Task Forces (USPSTF) for women at risk for developing perinatal depression [34]. Most studies evaluating interventions to prevent perinatal depression have used an indicated prevention approach, including individuals at increased risk for depression as determined by elevated depressive symptoms or a history of depression [34]. In addition, due to the high comorbidity between anxiety and depression [7], some studies aimed at preventing perinatal depression have assessed perinatal anxiety as a secondary outcome [34]; however, it is unclear to what extent interventions developed to prevent depression may also prevent perinatal anxiety. In this regard, it should be noted that the decrease in anxiety levels may be due to an indirect effect by improving depressive symptoms, without having a direct effect on anxiety.

Therefore, it is necessary to dedicate resources to the prevention of perinatal anxiety, even more so knowing its high prevalence and the consequences it entails for mothers and children. Knowing what type of preventive interventions are most frequently applied, the aspects they address, and which are the most effective would allow the development and implementation of preventive psychological interventions to reduce the probability of suffering anxiety at this stage of life.

So far, there have been few efforts aimed at preventing perinatal anxiety. Moreover, previous reviews have looked at interventions aimed at addressing anxiety and depression together [35], included both therapeutic and preventive interventions [36], have focused exclusively on preventing anxiety disorders [37], have analyzed a particular time in the perinatal stage such as pregnancy [38], or have focused on a specific therapeutic approach [29,39]. Therefore, there is a lack of studies that have focused on learning about existing psychological interventions to prevent or reduce the occurrence of both elevated anxious symptomatology and an anxiety disorder throughout the perinatal period. This review fills a gap in knowledge on this topic, as it is the first to focus on identifying psychological interventions aimed at preventing perinatal anxiety.

Knowing what preventive psychological interventions exist, their therapeutic approach, their components, and their efficacy may contribute to the development of appropriate interventions to address perinatal anxiety. This would contribute to avoiding its possible consequences for both mothers and their children. To this end, a systematic review of the research published on this subject in the last 10 years was carried out.

Based on the aforementioned, the main objective of this review was to know which psychological interventions have been applied for the prevention of perinatal anxiety. The specific objectives were to analyze (1) the most commonly used therapeutic approach and format; and (2) the type of interventions that have proven to be most effective.

## 2. Methods

The study’s selection process was carried out following recommendations of the Preferred Reporting Items for Systematic reviews and Meta-Analyses (PRISMA) guidelines [40]. Articles had to meet the following inclusion criteria: (1) be published in a scientific journal; (2) be written in English, but conducted in any country; (3) apply a psychological intervention with the aim of preventing or reducing perinatal anxiety; (4) the intervention had to be implemented during pregnancy or in the postpartum period; (5) evaluate the effect of the intervention quantitatively; (6) participants could not meet diagnostic criteria for a generalized anxiety disorder; and (7) published between March 2015 and March 2025. Additionally, studies evaluating pregnancy-specific anxiety were excluded, since it is known that it is a different entity from the diagnostic and continuous measures of general anxiety, as it is a specific type of anxiety characterized by fears and/or concerns about pregnancy [41]; those that assessed anxiety in conjunction with other emotional symptomatology (e.g., comorbidity between anxiety and depression, stress); those focused on specific anxiety disorders (e.g., obsessive compulsive disorder and post-traumatic stress disorder); studies including non-representative samples (e.g., twin pregnancies, families without resources); and other reviews.

No large-language models or AI tools were used in the preparation of this manuscript.

### 2.1. Search Strategy

A literature search was carried out in the PsycINFO, Medline, and SCOPUS databases to identify papers published between the specified period (31 March 2015 to 31 March 2025). The following keywords were used in the initial search by combining them as follows: “psychological intervention” OR “prevention” (abstract/title) AND “perinatal anxiety” OR “prenatal anxiety” OR “postnatal anxiety” (abstract/title). Reference lists of retrieved articles were also examined.

### 2.2. Strategy for the Selection of Studies and Analysis of Results

**Figure 1:**
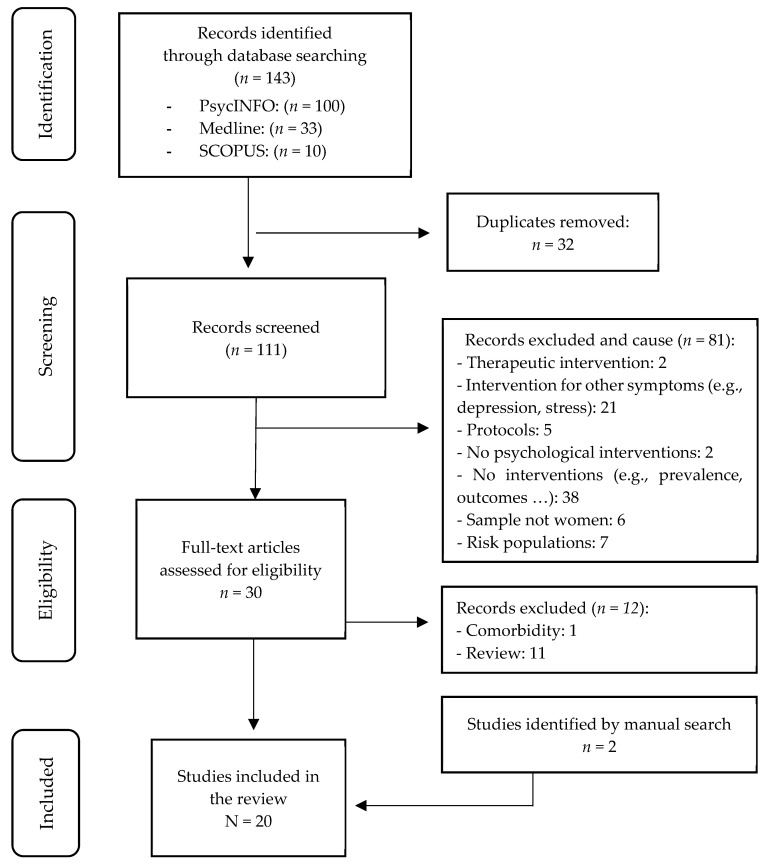
Flowchart of study selection.

Article selection was performed independently by two investigators and, in case of discrepancies, discussed between them. When no agreement was reached, the third author decided whether the article should be included. First, all publications found within the search criteria were transferred to Refworks and all repeated publications were removed. Subsequently, the studies were selected by reading the titles and abstracts, and finally the full text. The complete selection process is summarized in Figure 1. The extracted data were synthesized by year, objective, sample, measures, results, and conclusions. Statistical data from the studies were evaluated.

## 3. Results

### 3.1. Study Selection

The search strategy designed resulted in a total of 143 potential scientific publications: PsycINFO (*n* = 100), Medline (*n* = 33), and SCOPUS (*n* = 10). Of these, 32 were duplicates (Table S1), so the final number of publications after eliminating duplicates was 111.

A systematic search was then carried out in which the retrieved studies were screened. A series of inclusion and exclusion criteria were applied to guide the selection process. After reading the title and abstract of the studies, the investigators excluded a total of 81 studies for (1) being therapeutic interventions, i.e., for women who met the criteria for a mental disorder; (2) being interventions aimed at other symptomatology (e.g., depression, stress, distress); (3) being protocols; (4) not being psychological interventions; (5) addressing aspects other than an intervention (e.g., prevalence, consequences); (6) not being psychological interventions; (7) addressing aspects other than an intervention (e.g., prevalence, consequences); (8) not being psychological interventions; and (9) not addressing aspects other than an intervention (e.g., prevalence, consequences); (10) the sample was not women; and (11) they were at-risk populations (e.g., low-income women, battered women, etc.).

After selection, the full text of each of the 30 selected articles was analyzed and, finally, 18 studies were included. The other 12 were excluded because they addressed comorbidity between anxiety and depression (*n* = 1) or because they were systematic reviews (*n* = 11). Two other studies identified by a hand search were added. Thus, 20 articles were finally included in the review.

The quality of the selected studies was assessed using the Mixed Methods Assessment Tool (MMAT) [42] through two general screening questions and five specific questions, depending on the design of each study (in this case, quantitative randomized controlled trials and quantitative non-randomized studies). The researchers re-read the full text of each article, extracting the information of interest/relevance for the review. The selected results were then compiled in table format (Table S2). Based on the data provided by MMAT, it can be observed that, at a general level, the studies included in the review show a low risk of bias, allowing robust conclusions to be drawn in relation to psychological interventions to prevent perinatal anxiety.

### 3.2. Characteristics of Selected Studies

Twenty articles were reviewed that applied a psychological intervention to prevent or reduce anxiety during pregnancy or postpartum. Most of the interventions were applied in pregnancy (*n* = 18) and only two were applied in the postpartum. Australia and China were the countries with the most interventions (*n* = 5 each), followed by the Netherlands (*n* = 3) and Iran (*n* = 2). Romania, the USA, the UK, India, and Canada each contributed one study.

Regarding the type of design employed in each investigation, most were randomized controlled trials (*n* = 15), two were quasi-experimental studies, and three were pre-post designs. Great variability was observed in the sample size of each study ranging from 29 [43] to 529 women [44]. The total number of women included in the studies was 2889, being 2617 pregnant women and 272 postpartum women.

Several questionnaires were used to assess anxiety. Specifically, nine studies evaluated anxiety with the Generalized Anxiety Disorder Scale (GAD-7), being the most commonly used instrument. Five studies used the State-Trait Anxiety Inventory (STAI), two the Depression, Anxiety, and Stress Scale (DASS) and the Perinatal Anxiety Screening Scale (PASS). The Beck Anxiety Inventory (BAI), the Hamilton Anxiety Scale (HAM-A), the Anxiety Subscale of the Hospital Anxiety Depression Scale (HADS-A), and the Anxiety Subscale of the EPDS (EDS-3A) were employed in one study each.

Regarding who delivered the interventions, all were applied and/or guided by mental health professionals (psychologists/psychiatrists) or other trained professionals (e.g., midwives, meditation teachers).

With regard to ethical aspects, all studies included in the review had a data protection protocol in place, which guaranteed the confidentiality of participants’ personal data. Furthermore, women were required to sign an informed consent form before participating in the study. Regarding online interventions, women were given a password to access the sessions in order to preserve their anonymity.

### 3.3. Characteristics of Interventions

#### 3.3.1. Type of Prevention Program

Of the 20 interventions reviewed, eight were universal prevention interventions (Table 1) and 12 were indicated prevention interventions (Table 2). All universal prevention interventions [43,44,45,46,47,48,49,50] were delivered during pregnancy. Regarding indicated prevention, 10 interventions were conducted in pregnancy [51,52,53,54,55,56,57,58,59,60] and two [61,62] were applied in the postpartum.

#### 3.3.2. Therapeutic Approach and Application Format

In terms of the therapeutic approach applied, nine interventions employed mindfulness, eight employed cognitive behavioral therapy, one employed peer support therapy, one employed attentional bias training and one combined several therapeutic approaches (see Table 1 and Table 2).

Regarding the type of prevention applied, four of the universal interventions used cognitive behavioral therapy (CBT), two used mindfulness, one used peer support therapy and one used attentional bias training. Regarding the indicated prevention interventions, seven interventions employed mindfulness or mindfulness-based CBT, four employed CBT, and one combined several therapeutic approaches.

Of the 18 interventions implemented in pregnancy, seven employed mindfulness-based interventions [49,50,55,56,57,58,59,60] and two employed cognitive behavioral therapy-based mindfulness [54,58]. CBT was employed in seven interventions [44,45,47,48,51,52,53]. It should be noted that Anton and David [45] applied Rational Emotive Therapy (RET) and Sharma et al. [48] progressive muscle relaxation. On the other hand, Fontein-Kupiers et al. [46] conducted a peer support program where strategies and support resources were provided to prevent maternal distress. Finally, in the study by Dennis-Tiwary et al. [43], an attentional bias training program (ABMT) was applied, which consisted of training attention away from threat, i.e., redirecting attention away from stimuli perceived as negative or threatening, with the aim of reducing attentional bias towards threat.

In postpartum, Appleton et al. [61] combined different therapeutic approaches (CBT, mindfulness and self-compassion) and Loughnan, Butler et al. [62] applied CBT via internet.

In terms of implementation format, five of the universal programs were carried out face-to-face and three were developed online. With respect to the indicated prevention programs, seven interventions were carried out online and five were face-to-face.

Of the 18 interventions implemented in pregnancy, nine were delivered face-to-face, of which five were mindfulness interventions [49,50,54,55,58] and four were CBT [45,47,48,51]. The remaining nine interventions [43,44,46,52,53,56,57,59,60] were conducted online, via platforms or apps. Regarding postpartum, one intervention was delivered face- to-face [61] and one online [62].

A large proportion of the interventions (*n* = 9) were conducted in a group format [45,49,50,51,54,55,58,61]. However, it should be noted that Anton and David [45] conducted the first individual session and Sharma et al. [48] do not specify the application format. The online interventions were conducted individually.

The number of sessions used ranged from 4 [47,48,49,53,56,57,60] to 10–14 sessions [51] in pregnancy, with a duration of four sessions being most common (*n* = 7). In postpartum, the recommended number of sessions to complete the online program was six [62] and the Appleton et al. [61] program consisted of eight sessions.

#### 3.3.3. Effectiveness of Intervention

Of the 20 interventions analyzed (see Table 3), 12 [44,45,46,47,48,49,53,55,58,59,61,62] found significant reductions in anxiety levels in post-treatment assessments relative to those conducted at pre-treatment. Of these, six were universal prevention interventions [44,45,46,47,48,49] and six were indicated prevention [53,55,58,59,61,62].

Regarding the 18 interventions in pregnancy, 10 were effective in reducing anxiety. Of these, five were cognitive behavioral therapy interventions [44,45,47,48,53], four mindfulness [49,55,58,59], and another a peer support program [46]. Most (*n* = 6) were delivered face-to-face [45,47,49,55,58] versus online format [44,46,53,59]. Six interventions were conducted in groups [45,47,49,55,58,61] and four were developed individually [44,46,53,59]. In terms of the number of sessions of the interventions that were effective, four were comprised of four sessions [47,48,49,53], three had eight sessions [45,55,58], in one the number of sessions ranged from four to six [44], and one did not specify the number of sessions [46].

Regarding postpartum, the two interventions found that both CBT applied online [62] and psychotherapy developed face-to-face [61] proved to be effective in reducing anxiety levels. The number of sessions ranged from six to eight.

## 4. Discussion

### 4.1. Implications of the Study

One of the objectives of this review was to find out what type of psychological interventions have been most frequently applied to prevent or reduce anxiety during pregnancy and postpartum. It was found that indicated preventive interventions, that is, those aimed at reducing moderate or severe anxious symptomatology, were the most common in both pregnancy and postpartum. This is consistent with findings in other reviews investigating the efficacy of psychological interventions to address anxiety or anxiety disorders in the perinatal stage [36,37]. While early arrest of anxiety symptoms in the perinatal stage may help prevent the development of more serious mental health problems [63], most efforts have been aimed at reducing anxiety when it was already present. On the other hand, in this review it was observed that most of the interventions aimed at preventing perinatal anxiety were carried out during pregnancy (*n* = 18). These findings are in agreement with those found in other reviews [29,36,37], and they show us how little attention has been paid to the prevention of anxiety during postpartum. On the other hand, the advantage of performing psychological interventions during pregnancy is that they could be integrated into maternal education classes, which would reduce the associated stigma and costs [33]. Also, the benefits of preventing anxiety would extend to the baby before birth and would reduce the potential adverse consequences of experiencing anxiety during pregnancy for women, such as increased risk of postpartum depression. In addition, during gestation, women frequently seek health services and are more willing to receive help because they believe it will have a positive impact on their baby [18].

Regarding the therapeutic approach applied, mindfulness and CBT were the most employed approaches, although CBT was the one most associated with a significant reduction in anxiety levels. This is consistent with findings from other reviews where a greater number of interventions applying mindfulness have been observed [38], but a greater efficacy of CBT [36]. Our findings are also in line with the conclusions of the National Institute for Health and Clinical Excellence [64] and Maguire et al. [8] who note that CBT is an effective intervention for addressing anxiety during the perinatal stage. Likewise, they agree with the recommendations of NICE [64] and the National Collaborating Centre for Mental Health [65], which state that CBT should be the first-line treatment for addressing perinatal anxiety.

Regarding the format of application, it was observed that face-to-face interventions (*n* = 10) were as common as online interventions (*n* = 10). However, a greater number of interventions applied in the face-to-face format (*n* = 7) demonstrated their efficacy in reducing anxiety compared to the online format (*n* = 5). In this regard, it would be interesting to have studies comparing the efficacy of interventions according to the application format in order to draw reliable conclusions. It should be noted that during the perinatal stage interventions delivered via the Internet represent a tool with great potential [66], as they are usually more attractive and reduce the need to travel, one of the barriers associated with low access to treatments for psychological problems during this period [67]. Therefore, interventions developed online could be useful for preventing anxiety given their accessibility, anonymity, and cost-effectiveness [68].

Regarding the modality of application, most of the interventions were developed in a group setting (*n* = 9), and this format was also the one that showed the greatest efficacy (*n* = 6). This is interesting, since group interventions involve lower cost, high satisfaction for participants, and high compliance rates [63]. With respect to duration, it was found that the most frequent interventions consisted of four sessions (*n* = 7). Likewise, they were also the most effective. This seems to indicate that with brief interventions it would be possible to prevent perinatal anxiety. At the same time, having brief interventions would help facilitate adherence to them, since we are at a time in a woman’s life when one of the limitations to attending therapy is lack of time.

Finally, we believe it is important to highlight that regardless of the timing of the intervention, the type of preventive program, the therapeutic approach used, the delivery format, and the effectiveness of the intervention, it would be interesting to include in all interventions strategies aimed at addressing variables that have been shown to influence the development of perinatal anxiety. For example, it has been observed that women’s social or healthcare support can be a protective factor against perinatal anxiety [69]. Therefore, including strategies to improve social support in interventions could be beneficial. Another important aspect could be to include stress management strategies, as stress is often a risk factor for perinatal anxiety due to the multiple problems that can arise during pregnancy, such as miscarriage, stillbirth, or during the postpartum period, such as premature death, and difficulties in breastfeeding [70,71,72].

### 4.2. Limitations

Although, to our knowledge, this is the first review that analyzes preventive psychological interventions to address anxiety during pregnancy and postpartum, some limitations found should be pointed out. First, the existing controversy regarding the definition of anxiety is an important aspect to take into account, as the findings may vary depending on what each assessment instrument understands as anxiety. Also, the variability between the self-report instruments used and their cut-off points makes it difficult to interpret the results that may vary depending on what each assessment instrument understands as anxiety. Second, the review is limited to studies published in English, so the generalization of the findings is limited. Another limitation has to do with methodological aspects, such as the small sample size of some studies, [43,45], or the absence of a control group [44,49,55,61], and the scarcity of long-term follow-ups, which makes it difficult to generalize the results found.

The variability in the assessment instruments used and the shortcomings at the methodological level hinder the possibility of performing a meta-analysis of the findings, which could have provided additional information on the most effective type of intervention in terms of approach, duration, and application format. Another limitation was that publication bias was not assessed for the articles, as some of them did not meet the criteria required to perform a meta-analysis or complete Edger’s regression, which would have provided greater certainty about the results found. Likewise, the scarcity of interventions applied in the postpartum period makes it impossible to draw conclusions and extrapolate the results to the postpartum population. Additionally, this review excluded studies prior to 2015 and those based on high-risk populations (e.g., studies conducted on women with living in poverty situations, experiencing high-risk or twin pregnancies, or being victims of gender-based violence). Therefore, the generalization of the findings to these populations may be limited.

### 4.3. Future Research Directions

Future randomized controlled trials are required to evaluate the efficacy of preventive interventions during the perinatal stage, especially those aimed at universal prevention and during the postpartum period, as these are the interventions and the perinatal period that have been least researched. Another interesting methodological aspect would be to carry out long-term follow-up, as this would provide valuable information on which interventions maintain their efficacy over time.

## 5. Conclusions

This review provides updated knowledge on the preventive psychological interventions that have been carried out to address anxious symptomatology during the perinatal stage. The results show that the most common interventions are indicated prevention interventions, as well as that more attention has been given to the prevention of anxiety during pregnancy (see Table 3). The therapeutic approach that has been shown to be most effective is CBT. In addition, the results support a wide variety of delivery modes, including face-to-face, online, group, and individual, suggesting that psychological interventions can be tailored to the individual needs of women during this period. Given the negative consequences of untreated perinatal anxiety for mothers and children and women’s preference for psychological interventions, the present review argues for the availability and use of such interventions in the perinatal stage. Accordingly, it would be important to have mental health professionals to accompany women at this time in their lives. This would allow for more specialized care and devote more time and resources to perinatal mental health care, mitigating the possible consequences that may arise from it.

## Figures and Tables

**Table 1:** Universal prevention interventions.

Study	Sample	Duration	Intervention	Results					Conclusions
* Anton and David (2015) Romania [45]	48 pregnant women with no previous mental illness and no previous psychotherapy. IG: 23 CG: 25	9 weekly sessions of 90 min each. First individual session. Subsequent group sessions.	GI: 8-session REBT program GC: usual care	**Pretest**		**Half Treatment**	**Post-Test**		IG significantly reduces anxiety, depression and negative emotionality at mid-treatment and at the end of treatment. Differences in IG remain significant in anxiety and negative emotionality at 3 months postpartum.
				STAI-S (M)		(5th week)	(end)	3PPM	
				**IG**	42.22	39.11	35.58	32.46	
				**CG**	44.60	44.60	41.38	39.40	
* Fontein-Kuipers et al. (2016) Netherlands [46]	433 pregnant women (4–14 weeks gestation). IG: 218 CG: 215	Onset in the 1st trimester of pregnancy.	IG: online support program “WazzUp Mama?!” CG: usual care		**Pretest** (1st trimester)		**Post-Test** (3rd trimester)		There were significant reductions in anxiety levels between the first and third trimester. The results support the efficacy of the intervention.
					STAI-T ≥ 41				
				**IG**	28.7		26.9		
				**CG**	28.9		31.6		
* Salehi et al. (2016) Iran [47]	91 nulliparous pregnant women (13–26 weeks gestation) with a low to moderate level of anxiety (STAI < 75). IG1: 31 IG2: 30 CG: 30	Beginning in the 2nd trimester of pregnancy. 4 sessions (2 sessions/week) of 120–150 min.	IG^1^: group cognitive behavioral therapy (GCBT). IG^2^: interactive classes. CG: usual care		**Pretest**		**Post-Test** (4 weeks after treatment)		CBT and interactive lectures were effective in reducing both state and trait anxiety at 4 weeks after the intervention compared to the control group. CBT was more effective than lectures.
					STAI-T (M)				
				**IG^1^**	38.5		32.3		
				**IG^2^**	40.8		36.2		
				**CG**	37.7		38.0		
					STAI-S (M)				
				**IG^1^**	38.0		38.0		
				**IG^2^**	41.1		41.1		
				**CG**	38.3		38.3		
Dennis-Tiwary et al. (2017) USA [43]	29 women (19–29 weeks of gestation). IG: 15 CG: 14	Complete 160 rounds (40/week) of 40 min.	IG: attention bias modification training (ABMT). CG: placebo training (PT).		**Pretest**		**Post-Test** (end treatment)		It was observed that there was an increase in anxiety levels between the pre- and post-test means in the intervention group, as assessed using the DASS and HAM-A.
					DASS (M)				
				IG	2.87		3.20		
				CG	2.15		2.07		
					HAM-A (M)				
				IG	7.73		9.20		
				CG	7.92		6.93		
* Warriner et al. (2018) UK [49]	64 pregnant women.	4 weekly sessions of 2.5 h (10 h)	IG: Mindfulness-based childbirth and parenting (MBCP). It combines traditional prenatal teaching with mindfulness-based skills.	**Pretest**		**Post-Test** (2 weeks)			Significant differences were observed between pre- and post-test measures for anxiety, depression, stress and worry. This prenatal intervention shows promise for implementation in the national health care system.
				GAD-7 (M)					
				8.53		5.08			
Zhang et al. (2019) China [50]	63 women between 14 and 28 weeks of gestation. IG: 32 CG: 31	8 weekly sessions of 90 min.	IG: Mindfulness-based stress reduction (MBSR) Group therapy (3 to 6 women). CG: usual care.			**Pretest**	**Post-Test**		This study provides preliminary evidence that MBSR is suitable for decreasing prenatal stress and anxiety in pregnant Chinese women.
						STAI (M)			
				**IG**		71.53	66.91		
				**CG**		71.29	72.13		
* Sharma et al. (2020) India [48]	70 pregnant women ≥ 20 weeks gestation. IG: 35 CG: 35	4 sessions.	IG: progressive muscle relaxation technique (PMRT). CG: conventional care.			**Pretest**	**Post-Test** (4 weeks)		The progressive muscle relaxation technique is effective in reducing anxiety in pregnant women after 4 weeks of intervention.
				PASS (M)					
				**IG**		33.82	28.88		
				**CG**		34.28	38.45		
* Mahoney et al. (2023) Australia [44]	529 pregnant women.	3 online skills training lessons. Recommended to be completed in 4–6 weeks. Available for 12 months.	IG: Internet-applied cognitive behavioral therapy (iCBT).	**Pretest**		**Post-Test**			Significant pre- and post-treatment reductions in symptoms of anxiety, depression and psychological distress were observed. The results support the use of iCBT in the perinatal stage.
				GAD-7 (EMM)					
				9.62		6.76			

STAI-S: state anxiety subscale of the STAI; REBT: Rational Emotive and Behavioral Therapy; STAI-T: trait anxiety subscale of the STAI; DASS: Depression, Anxiety, and Stress Scale; HAM-A: Hamilton Anxiety Scale; PASS: Perinatal Anxiety Screening Scale; GAD-7: Generalized Anxiety Disorder; IG: intervention group; CG: control group; M: mean; EMM: estimated Marginal Mean; *: studies that have shown statistically significant differences (*p* < 0.05).

**Table 2:** Indicated preventive interventions.

Study	Sample	Duration	Intervention	Results							Conclusions
**Pregnancy**											
* Yazdanimehr et al. (2016) Iran [58]	63 women between the first and sixth month of gestation and with EPDS > 13 BAI > 16 IG: 30 CG: 33	8 sessions of 90 min	IG: cognitive behavioral therapy based on mindfulness. CG: usual care. At the end of the intervention, they received a manual on the sessions given in the IG.			**Pretest**	**Post-Test**				Mindfulness-based cognitive behavioral therapy can significantly reduce anxiety in pregnant women.
							(end)		(1 month)		
				BAI (M)							
				**IG**		M = 19.76	M = 10.86		IG		
				**CG**		M = 20.24	M = 20.54		CG		
* Townshend et al. (2018) Australia [55]	109 women < 30 weeks gestation, having had a previous episode of depression and/or elevated depression scores, having risk factors for anxiety or perinatal depression.	8 sessions (1 session per week) 30-min home practice.	IG: Caring for Body and Mind in Pregnancy (CBMP)	**Pretest**			**Post-Test**				CBMP significantly reduced perinatal anxiety and general stress scores.
				DASS-21 (M)							
				9.99			8.49				
				PASS (M)							
				33.95			27.13				
* Loughnan, Sie, et al. (2019) Australia [53]	77 women (13–30 weeks gestation) who met criteria for probable GAD and/or major depression and internet access. IG: 43 CG: 44	IG: Access to a virtual platform in which they had to complete three skills training lessons in 4 weeks. They did not have to go to their health center except if there was suicidal ideation. CG: they could make use of health services. At the end of the study they had access to the MUMentum program.	IG: internet-applied brief cognitive behavioral therapy (iCBT): MUMentum Pregnancy. CG: usual care.			**Pretest**	**Post-Test**				Significant differences were observed between IG and CG in the reduction in anxiety at 1 week after the intervention, but not at 1 month. IG significantly reduced anxiety.
							(1 week)		(4 weeks)		
				GAD-7 (M)							
				**GI**		12.66	7.49		GI		
				**GC**		11.84	9.43		GC		
Yang et al. (2019) China [56]	123 women between 20–34 weeks gestation with GAD-7 or PHQ-9 > 4 and with internet access. IG: 62 CG: 61	The intervention was carried out through a virtual platform. It consisted of 4 sessions of 40 min each, developed over 8 weeks. There was also a telematic group for participants to share experiences.	IG: online therapy based on mindfulness. CG: usual care combined with emotion management skills.			**Pretest**	**Post-Test** (end)				Significant reductions in intergroup and IG anxiety and depression were found at the end of the intervention. Mindfulness interventions during pregnancy could be part of the psychological care provided to women during pregnancy.
						GAD-7 (M)					
				**IG**		5.52	2.97				
				**CG**		5.19	5.26				
Burger et al. (2020) Netherlands [51]	282 pregnant women with moderate or severe anxiety and/or depression scores. IG: 140 CG: 142	Beginning in the 20th week of gestation and ending 3 months after delivery. 10–14 individual sessions.	IG: cognitive behavioral therapy CG: usual care			STAI (M)					No improvement was found in the levels of anxiety and depression at any time after the intervention. CBT applied in the perinatal stage does not improve emotional symptomatology.
						**IG**	**CG**				
				**Pretest**		48.6	48.6				
				24 SG		47.7	47.7				
				36 SG		43.2	43.2				
				6 SPP		40.9	40.9				
				3 MPP		43.8	43.8				
				6 MPP		42.1	42.1				
				12 MPP		41.0	41.0				
				18 MPP		40.9	40.1				
Heller et al. (2020) Netherlands [52]	159 women < 30 weeks gestation with CES-D> 16 and/or HADS-A≥ 8. IG: 79 CG: 80	Start before 30 weeks of gestation. IG: five modules presented on a weekly basis. Each module consists of information, examples of other mothers and homework.	IG: internet-based intervention “MamaKits online” based on problem-solving. CG: usual care	**Pretest**			**Post-test**				No significant differences were found between the groups.
				**HADS-A** **(>8)**		End	36 GW		6 PPW		
				**IG**	11.4	8.4	7.9		7.1		
				**CG**	11.9	8.6	7.9		7.9		
MacKinnon et al. (2021) Canada [54]	60 pregnant women between 12–28 weeks of gestation with elevated levels of distress. IG: 28 CG: 32	IG: 8 group sessions (3–6 participants) per week of 2 h duration. CG: usual care.	Mindfulness-based cognitive therapy for perinatal depression (MBCT-PD).	* Point out the quadratic effect.							The intervention group significantly reduced levels of distress, but there was no reduction in symptoms of depression and anxiety.
							**Pretest**				
				GAD-7 (M)							
				**IG**			6.32				
				**CG**			8.52				
Yang et al. (2022) China [57]	149 women with gestational age < 32, with GAD-7 or PHQ-9 > 4 and with internet access. IG: 100 MT = 50 MAT = 50 CG: 49	The intervention was carried out through a virtual platform. The participants received 4 weekly mindfulness sessions lasting an hour and a half. There was also a telematic group to share experiences among the participants. During weeks 2 and 4 the psychologist contacted them to solve problems and encourage adherence. MT: present-centered training MAT: acceptance-centered training.	IG: online therapy based on mindfulness-based mindfulness focused on the present (MT). IG: online therapy based on mindfulness focused on acceptance (MAT). CG: course of emotion management without mindfulness practice.				GAD-7 (M)				Significant differences in anxiety and depression were observed in the MAT group. Differences were not significant in the MAT group nor in the control group, nor between both groups. Mindfulness could be a promising technique to improve anxious and depressive symptoms.
				**Pretest**		**MT**	6.96				
						**MAT**	7.20				
						**CG**	6.90				
				**Post-test** (2 weeks)		**MT**	5.48				
						**MAT**	2.60				
						**CG**	6.50				
* Zhang, Li et al. (2023) China [59]	160 women between 12–24 weeks gestation with symptoms of anxiety (GAD-7 ≥ 5) or depression EPDS ≥ 9). IG: 80 CG: 80	The intervention was carried out through a Wechat program. It consisted of 6 modules, each lasting 1 week. Each module consisted of a thematic lesson and homework.	IG: guided self-help mindfulness therapy (Wechat). CG: usual care.		Pre (12–20 GW)	End (20–28 GW)	Before delivery (36–37 GW)	6PPW	3PPM	6PPM	Digitally delivered programs are effective in reducing distress among pregnant women.
					GAD-7 (M)						
				**IG**	5.56	3.14	3.32	4.49	4.34	3.75	
				**CG**	5.80	5.61	6.18	7.31	5.90	5.90	
Zhang, Lin et al. (2023) China [60]	108 women between 12 and 24 weeks of pregnancy with symptoms of anxiety (GAD-7 ≥ 5) or depression (EPDS ≥ 9). IG: 54 CG: 54	IG: Intervention carried out through the WeChat program. It consisted of four weekly sessions, each lasting 30 min, and between 30 and 45 min of daily mindfulness practice. CG: Education on prenatal care through the WeChat program.	IG: Mindfulness course through the WeChat program. CG: prenatal education.			**Pretest**	**Post-Test**				The self-help and mindfulness intervention reduced prenatal stress and negative affect and improved positive affect and mindfulness.
							(end treatm.)		(15 weeks)		
				GAD-7 (M)							
				**IG**		5.73	4.56		**IG**		
				**CG**		5.63	5.98		**CG**		
**Postpartum**											
* Loughnan, Butler, et al. (2019) Australia [62]	131 women within 12 months postpartum with GAD-7 and/or PHQ-9 ≥ 10 and with internet access. IG: 69 CG: 62	IG: Access to a virtual platform where they had to complete three skill training lessons in six weeks. They were only contacted if they presented suicidal ideation. CG: They could make use of health services.	GI: Brief Cognitive Behavioral Therapy applied via the internet (iCBT): MUMentum Postnatal CG: usual CARE			**Pretest**	**Post-Test**				Significant reductions in intergroup and intragroup anxiety and depression levels were observed both one week and one month after the intervention. There is preliminary evidence of the program’s effectiveness in treating anxiety and/or depression in women during the postpartum period.
							(1 week)		(4 weeks)		
				GAD-7 (M)							
				**IG**		12.08	6.66		**GI**		
				**CG**		12.26	10.44		**GC**		
* Appleton et al. (2025) Australia [61]	141 women up to 24 weeks postpartum who score high in stress, depression or anxiety.	IG: Eight weekly two-hour (16 h) group sessions per week with a maximum of 12 women per group and two facilitators + a follow-up session (group meeting) four weeks after the last sessions, and an individual psychoeducation session for the woman’s partner around the fifth or sixth week after the last sessions, with a maximum of 12 women per group and two facilitators + a follow-up session (group meeting) four weeks after the last sessions, and an individual psychoeducation session for the woman’s partner around the fifth or sixth week after the last sessions.	IG: Group psychotherapy program	**Pretest**			**Post-Test**				These findings support the efficacy of group psychotherapy interventions in reducing symptoms of depression and anxiety in the postpartum period.
				EDS-3A (M)							
				5.68			4.37				

BAI: Beck Anxiety Inventory; DASS: Depression, Anxiety, and Stress Scale; PASS: Perinatal Anxiety Screening Scale; GAD-7: Generalized Anxiety Disorder; STAI: Spielberg State-Trait Anxiety Inventory; HADS-A: Anxiety Subscale of the Hospital Anxiety Depression Scale; GW: Gestational Week; PPW: Postpartum Week; PPM: Postpartum Month; EPDS: Edinburgh Postnatal Depression Scale; EDS-3A: Anxiety Subscale of the EPDS; IG: intervention group; CG: control group; M: mean; EMM: estimated Marginal Mean; *: studies that have shown statistically significant differences (*p* < 0.05).

**Table 3:** Summary of the main characteristics of the selected studies.

Study	Intervention Type	Perinatal Period (Pregnancy/Postpartum)	Therapeutic Approach	Application Format	Effective Interventions
Anton & David (2015) [45]	Universal	Pregnancy	CBT	Face-to-face Individual + Group	V
Fontein-Kuipers et al. (2016) [46]	Universal	Pregnancy	Peer support program	Online Individual	V
Salehi et al. (2016) [47]	Universal	Pregnancy	CBT	Face-to-face Group	V
Yazdanimehr et al. (2016) [58]	Selective/ Indicated	Pregnancy	Cognitive behavioral therapy based on mindfulness	Face-to-face Group	V
Dennis-Tiwary et al. (2017) [43]	Universal	Pregnancy	ABMT	Online Individual	
Townshend et al. (2018) [55]	Selective/ Indicated	Pregnancy	Mindfulness	Face-to-face Group	V
Warriner et al. (2018) [49]	Universal	Pregnancy	Mindfulness	Face-to-face Group	V
Loughnan, Sie, et al. (2019) [53]	Selective/ Indicated	Pregnancy	CBT	Online Individual	
Yang et al. (2019) [56]	Selective/ Indicated	Pregnancy	Mindfulness	Online Individual	
Zhang et al. (2019) [50]	Universal	Pregnancy	Mindfulness	Face-to-face Group	
Burger et al. (2020) [51]	Selective/ Indicated	Pregnancy	CBT	Face-to-face Group	
Heller et al. (2020) [52]	Selective/ Indicated	Pregnancy	CBT	Online Individual	
Sharma et al. (2020) [48]	Universal	Pregnancy	CBT	Face-to-face Not specified	V
MacKinon et al. (2021) [54]	Selective/ Indicated	Pregnancy	Cognitive behavioral therapy based on mindfulness	Face-to-face Group	
Yang et al. (2022) [57]	Selective/ Indicated	Pregnancy	Mindfulness	Online Individual	
Mahoney et al. (2023) [44]	Universal	Pregnancy	CBT	Online Individual	
Zhang, Li, et al. (2023) [59]	Selective/ Indicated	Pregnancy	Mindfulness	Online Individual	V
Zhang, Lin, et al. (2023) [60]	Selective/ Indicated	Pregnancy	Mindfulness	Online Individual	
Loughnan, Butler, et al. (2019) [62]	Selective/ Indicated	Postpartum	CBT	Online Individual	V
Appleton et al. (2025) [61]	Selective/ Indicated	Postpartum	Combines different therapeutic approaches	Face-to-face Group	V

## Data Availability

No new data were created or analyzed in this study. Data sharing is not applicable to this article.

## Supplementary Materials

The following supporting information can be downloaded at: https://www.mdpi.com/article/10.3390/brainsci15080861/s1, Table S1. Duplicate removal process using the RefWorks tool. Table S2. MAT: Quantitative Non-Randomized Studies.
