# Supplementary material for: Preventive Psychological Interventions for the Management of Perinatal Anxiety: A Systematic Review
Source: Brain Sci. 2025 Aug 13;15(8):861. doi: 10.3390/brainsci15080861 (PMC12384845; doi:10.3390/brainsci15080861)
Supplement: Supplementary file 1 [file brainsci-15-00861-s001.zip › brainsci-3754429-Table S1.pdf]

Suplemmentary Table S1. Duplicate removal process using the RefWorks tool.

Database	Articles identified in the initial search	Criteria for removing duplicates	Number of duplicates removed	Articles after removing duplicates
PsycINFO	100	Match between the title, author, and year of publication.	6	94
Medline	33		22	11
SCOPUS	10		3	7
	143		32	111
