# Supplementary material for: Preventive Psychological Interventions for the Management of Perinatal Anxiety: A Systematic Review
Source: Brain Sci. 2025 Aug 13;15(8):861. doi: 10.3390/brainsci15080861 (PMC12384845; doi:10.3390/brainsci15080861)
Supplement: Supplementary file 1 [file brainsci-15-00861-s001.zip › brainsci-3754429-Table S2.pdf]

Table S2. MMAT: Quantitative Randomized Controlled Trials.

STUDY	S1. Are there clear research questions?	S2. Do the collected data allow to address the research questions?	2.1. Is randomization appropriately performed?	2.2. Are the groups comparable at baseline?	2.3. Are there complete outcome data?	2.4. Are outcome assessors blinded to the intervention provided?	2.5 Did the participants adhere to the assigned intervention?
**Anton and David (2015)**	Yes	Yes	Yes	Yes	Yes	Yes	Yes
**Fontein-Kuipers et al. (2016)**	Yes	Yes	No	Yes	Yes	?	Yes
**Yazdanimehr et al. (2016)**	Yes	Yes	Yes	Yes	Yes	Yes	Yes
**Salehi et al. (2016)**	Yes	Yes	Yes	Yes	Yes	?	Yes
**Dennis-Tiwary et al. (2017)**	Yes	Yes	Yes	Yes	Yes	Yes	Yes
**Zhang et al. (2019)**	Yes	Yes	Yes	Yes	Yes	?	Yes
**Loughnan, Sie, et al. (2019)**	Yes	Yes	Yes	Yes	Yes	?	Yes
**Loughnan, Butler, et al. (2019)**	Yes	Yes	Yes	Yes	Yes	?	Yes
**Yang et al. (2019)**	Yes	Yes	Yes	Yes	Yes	Yes	Yes
**Burger et al. (2020)**	Yes	Yes	Yes	Yes	Yes	?	No
**Heller et al. (2020)**	Yes	Yes	Yes	Yes	Yes	?	No
**Sharma et al. (2020)**	Yes	Yes	No	Yes	Yes	?	Yes
**MacKinon et al. (2021)**	Yes	Yes	?	Yes	No	Yes	Yes
**Yang et al. (2022)**	Yes	Yes	Yes	Yes	Yes	Yes	Yes
**Zhang, Lin et al. (2023)**	Yes	Yes	Yes	Yes	Yes	Yes	Yes
**Zhang, Li et al. (2023)**	Yes	Yes	Yes	Yes	Yes	Yes	Yes

Note. ?: Can’t tell

Table S2. MMAT: Quantitative Non-Randomized Studies (Cont.).

STUDY	S1. Are there clear research questions?	S2. Do the collected data allow to address the research questions?	3.1. Are the participants representative of the target population?	3.2. Are measurements appropriate regarding both the outcome and intervention (or exposure)?	3.3. Are there complete outcome data?	3.4. Are the confounders accounted for in the design and analysis?	3.5 During the study period, is the intervention administered (or exposure occurred) as intended?
**Warriner et al. (2018)**	Yes	Yes	No	Yes	Yes	Yes	Yes
**Townshend et al. (2018)**	Yes	Yes	?	Yes	Yes	Yes	Yes
**Mahoney et al. (2023)**	Yes	Yes	Yes	Yes	Yes	Yes	Yes
**Appleton et al. (2025)**	Yes	Yes	Yes	Yes	Yes	Yes	Yes

Note. ?: Can’t tell

Contributors: Alba Val and Cristina M. Posse.

Hong, Q.N.; Pluye, P.; Fàbregues, S.; Bartlett, G.; Boardman, F.; Cargo, M.; Dagenais, P.; Gagnon, M.P.; Griffiths, F.; Nicolau, B.; et al. Mixed methods appraisal tool (MMAT). Version 2018. McGill University, 2018, http://mixedmethodsappraisaltoolpublic.pbworks.com/w/file/fetch/127916259/MMAT_2018_criteria-manual_2018-08-01_ENG.pdf
